# Drug Development and the Use of Induced Pluripotent Stem Cell-Derived Cardiomyocytes for Disease Modeling and Drug Toxicity Screening

**DOI:** 10.3390/ijms21197320

**Published:** 2020-10-03

**Authors:** Paz Ovics, Danielle Regev, Polina Baskin, Mor Davidor, Yuval Shemer, Shunit Neeman, Yael Ben-Haim, Ofer Binah

**Affiliations:** 1 Department of Physiology, Biophysics and Systems Biology, The Rappaport Institute, Ruth & Bruce Rappaport Faculty of Medicine, Technion, Haifa 31096, Israel; pazovics@gmail.com (P.O.); danielleregev1504@gmail.com (D.R.); polina19994@gmail.com (P.B.); davidormor@gmail.com (M.D.); yshemer@hotmail.com (Y.S.); shunit36@gmail.com (S.N.); 2 Institute of Molecular and Clinical Sciences, St. George’s University of London, London SW17 0RE, UK; yael@ben-haim.com; 3 Cardiology Clinical Academic Group, St. George’s University Hospitals NHS Foundation Trust, London SW17 0QT, UK

**Keywords:** iPSC-CMs, drug screening, Adverse Drug Reactions (ADRs), cardiotoxiciy, structural mutations, channelopathies, metabolic mutations, laminopathies

## Abstract

Over the years, numerous groups have employed human induced pluripotent stem cell-derived cardiomyocytes (iPSC-CMs) as a superb human-compatible model for investigating the function and dysfunction of cardiomyocytes, drug screening and toxicity, disease modeling and for the development of novel drugs for heart diseases. In this review, we discuss the broad use of iPSC-CMs for drug development and disease modeling, in two related themes. In the first theme—drug development, adverse drug reactions, mechanisms of cardiotoxicity and the need for efficient drug screening protocols—we discuss the critical need to screen old and new drugs, the process of drug development, marketing and Adverse Drug reactions (ADRs), drug-induced cardiotoxicity, safety screening during drug development, drug development and patient-specific effect and different mechanisms of ADRs. In the second theme—using iPSC-CMs for disease modeling and developing novel drugs for heart diseases—we discuss the rationale for using iPSC-CMs and modeling acquired and inherited heart diseases with iPSC-CMs.

## 1. Introduction

One of the major hurdles during the tremendously expensive and extensive process of drug development is the discovery of Adverse Drug reactions (ADRs) which may interrupt prematurely the agonizing journey of bringing a new chemical entity (NCE) to the market. A key task in drug development is to identify cardiotoxicity as one of the major factors responsible for discontinuing the process. As illustrated in Table 1, numerous drugs are associated with various cardiovascular side effects, such as electrophysiological disturbances (e.g., delayed- and early-afterdepolarizations and triggered arrhythmias) as well as contractile dysfunction, which may lead to life-threating arrhythmias and heart failure, respectively. Reports analyzing drug development failures between 2011 and 2015 showed that the second most common cause for failure was the lack of safety, with 24–28% of drugs found to be unsafe in clinical trials [1,2]. ADRs including cardiotoxicity can be discovered during the long drug development phase or, worse, after the drug was released to the market (i.e., post-marketing). This may cause a catastrophic clinical outcome to the patients, as well as a considerable economic burden to the insurance and healthcare systems.

To address key issues regarding the process of drug development, in vitro disease modeling and screening drug toxicity, this article is composed of two parts. In the first part, we focus on the process of drug development and address in depth the issues of ADRs, drug-induced cardiotoxicity, safety screening during drug development and different mechanisms underlying ADRs. The second part is focused on the use of human induced pluripotent stem cell-derived cardiomyocytes (iPSC-CMs) for disease modeling and drug discovery. Specifically, this part addresses the rationale for using iPSC-CMs and modeling acquired and inherited heart diseases with iPSC-CMs.

## 2. Drug Development, Adverse Drug Reactions, Mechanisms of Cardiotoxicity and the Need for Efficient Drug Screening Protocols

In the process of drug development, preclinical safety and pharmacokinetics assessments of candidate drugs to ensure the safety profile are critical. In view of the lengthy and costly process of bringing New Chemical Entities (NCE) to the market, removing a candidate drug as early as possible in the development process is essential and highly effective. As indicated by the US Food and Drugs Administration (Food and Drug Administration (2004) Challenge and opportunity on the critical path to new medical products.): “The inability to better assess and predict product safety leads to failures during clinical development and, occasionally, after marketing” [3].

### 2.1. The Formal Paths for New Drug Approval

**Figure 1:**
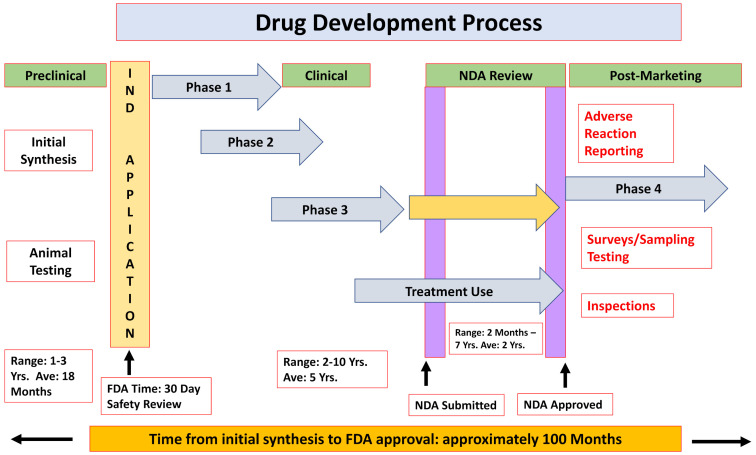
A scheme describing the drug development process. IND, Investigational New Drug; NDA, New Drug Application.

To acquaint the reader with this process, we present here the required steps for introducing a new drug to the market. The formal process is controlled by two main authorities: The Food and Drug Administration (FDA) in the USA and the European Medicines Agency (EMA) in Europe. Briefly, drug development includes three pre-marketing phases and activities and one post-marketing phase [4] (Figure 1).

❖ **Chemical and biological research**
  - Comprehensive understanding of the disease pathophysiology
  - Development of a NCE or repurpose an already-approved drug
  - Testing the drug effects/efficacy in in vitro and in vivo applicable models
❖ **Pre-clinical development**
  - Pharmacokinetics and pharmacodynamics characterization
  - Acute and late onset toxicology, teratogenicity, mutagenicity and carcinogenicity
  - Feasibility—proof-of-concept
❖ **Clinical trials**

These are the final, most critical and highly expensive stages, which are commonly divided into four Phases [5]. Phase 0: This phase is not mandatory for new drug development and is performed on 10–15 healthy volunteers in order to evaluate only the preliminary pharmacokinetic data. Phase 1: This is the first use of the drug in humans, for determining the safety profile. This phase includes 10–20 healthy volunteers or patients. Phase 2: The purpose of this phase is to evaluate the safety and efficacy of the drug (dose range/tolerance, maximal positive effect and minimal side effect) and is conducted on up to several hundred patients diagnosed with the disease to be treated. Phase 3: The goal of this phase, known as a confirmatory phase, is to verify safety and efficacy. This stage consists of one or more pivotal studies with the aim of demonstrating independent and reproducible results and includes hundreds to thousands of patients. Phase 4 and Post-Marketing surveillance: The goals of this phase are to establish a long-term safety profile in larger patient populations, discover potential different indications, analyze cost/benefit marketing and determine drug interactions and the efficacy and safety in different populations (e.g., elderly patients and patients with comorbidities that may not have been included in Phase 3).

### 2.2. Failure Rates of Drugs, Drug Development, Marketing and Adverse Drug Reactions (ADRs)

As noted by Arrowsmith and Miller, “Knowledge of the rates and causes of drug candidate attrition by clinical development phase and by therapeutic area is valuable in assessing the impact of changes in development strategy and research area focus by the pharmaceutical industry” [1]. During 2011–2012, there were a total of 148 failures between Phase 3 and submission, also including Phase 1/2 studies in patients and major new indications of already marketed drugs. Of these, 105 had reported reasons for failure; the majority were due to a lack of efficacy (56%) or to safety issues (28%), which include those failures due to an insufficient therapeutic index. In a more recent study, Harrison analyzed the reported causes of drug candidate attrition for the period 2013−2015 [2]. During this time period, there were 218 reported failures between Phase 2 (including Phases 1/2) and submission, where each drug indication combination is considered as a separate record and both new active substances and new indications of marketed drugs are included in the analysis). Of these failures, 174 had stated the reason for the failure and these were used in the subsequent analysis. Most of the failures were due to a lack of either efficacy (52%) or safety (24%), where safety includes those failures that were due to an insufficient therapeutic index. Strategic (15%), commercial (6%) and operational (3%) reasons were cited for the remainder of the failures. The breakdown by therapeutic area showed that the highest percentages of failure were in oncology (32%), central nervous system (CNS) diseases (17%) and musculoskeletal diseases (13%), with additional contributions from cardiovascular (7%), alimentary (7%), metabolic (6%) and infectious (5%) diseases. In Phase 2, the failures were primarily due to insufficient efficacy (48%) or safety (25%), and combined, these represent 73% of all Phase 2 failures. Similarly, insufficient efficacy was the primary reason for failure in Phase 3 (which provides the highest level of evidence that an experimental treatment is safe and efficacious [6]) (55%), followed by safety (14%). As indicated by the author, “although the decrease in late-stage failures for safety is encouraging, one would hope that for an industry becoming better at ‘failing early’, the proportion of compounds failing owing to insufficient efficacy would have been lower for phase III than for phase II, but this was not observed”. This decrease in failure rates between the Phases 2 and 3 raises the expectation that, due to improvements in early detection protocols, in the coming years, failure rates due to safety issues will continue to decrease.

The four Phases of clinical trials, in particular Phases 2 and 3, are more likely to detect ADRs that are common and with early onset. However, ADRs are detected in the post-marketing Phase as well. Between 1953 and 2013, 462 drugs were withdrawn from the market, with 63 (14%) due to serious cardiovascular ADRs [7] (Figure 1B). In a more recent review, Kocadal at el. reported that between 1950 and 2017, 464 drugs (53 of which had cardiovascular indications) were withdrawn from the market [8]. These included various categories such as psychostimulants (e.g., fenfluramine, sibutramine, benfluorex and dexfenfluramine), nonsteroidal anti-inflammatory drugs (NSAIDs) (e.g., valdecoxib, rofecoxib, alphacetylmethadol and parecoxib), antiarrhythmics (e.g., adenosine, bepridil and encainide) and antipsychotics (e.g., sertindole). In some cases, the ADRs were identified long after the drug was launched; for example, benfluorex, an anorectic and hypolipidemic drug, was removed from the market after 33 years of use, when it was found to be associated with valvular heart disease, estimated to have caused 500–2000 deaths [9,10]. Another example is bepridil (a calcium channel blocker used to treat angina) which was withdrawn from the market on 2004 after 23 years of use due to QT interval prolongation associated with Torsades de Pointes (TdP), first observed on 1982 [7]. Besides the serious clinical outcomes of ADRs, they inflict heavy financial costs. In this regards, Bond and Raehl studied over 8 million hospitalized patients and found that the combined yearly cost of ADRs, including hospitalization, medical monitoring and additional drugs used to treat drug toxicity, was ~300,000,000 USD [11]. The observation that a relatively high proportion of drugs fail due to safety issues during Phases 2 and 3 and during post-marketing, emphasizes the fact that drug safety screening and monitoring pre-marketing are presently insufficient, and that there is a critical need to create highly specific and sensitive human-compatible models that identify ARDs at all stages of drug development. These models are likely to reduce the enormous economical and clinical burden.

### 2.3. Drug-Induced Cardiotoxicity

**Figure 2:**
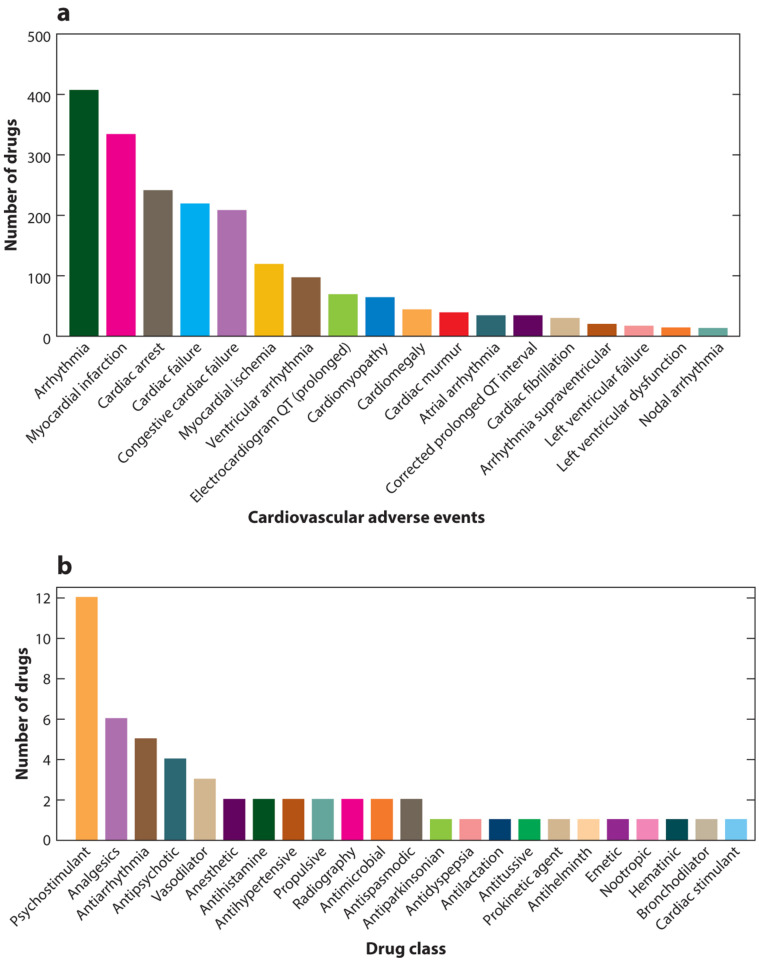
Cardiovascular ADRs and drugs indication causing ADRs. (**a**) On-market drugs and cardiovascular adverse events. Data adapted from the SIDER 4.1: Side Effect Resource (http://sideeffects.embl.de) database of drugs and adverse drug reactions. (**b**) Drug withdrawal due to serious ADRs. Sixty-three drugs in different therapeutic classes were withdrawn from the market between 1953 and 2013 owing to serious cardiotoxic effects [13].

The number of ADRs reported by the US Food and Drug Administration (FDA) Adverse Event Reporting System (FAERS) is constantly increasing, with 335,624 reports in 2006, 1,203,905 in 2014 and 2,191,808 in 2019 [12]. This is an alarming increase in ADRs, which constitutes a prominent cause for morbidity and mortality worldwide. Cardiac ADRs include major abnormalities such as ventricular depolarization and repolarization disturbances, QT shortening and prolongation, arrhythmias and heart failure. Cardiac ADRs may present both early as acute onset events shortly after receiving the drug, or delayed late onset events, presenting even years after exposure. Specifically, 1430 marketed drugs constituting a broad range of drug categories, with/without cardiovascular indications, have been associated with cardiovascular ADRs [13] (Figure 2A). Different drug categories were found to cause cardiovascular ADRs, with incidence as high as 35% for Tyrosine Kinase Inhibitors (TKIs) [14]. These adverse reactions may be dose-dependent, as is the case with Tricyclic Anti-Depressants (TCA), steroids and anthracyclines [15,16,17,18,19], or idiosyncratic, as with trastuzumab [20].

### 2.4. Safety Screening during Drug Development

During the past decade, several guidelines for preclinical and clinical cardiovascular ADRs screening were developed. The International Council for Harmonisation of Technical Requirements for Pharmaceuticals for Human Use (ICH) has developed safety guidelines consisting of two protocols—ICH S7B and ICH E14 [21,22]. The ICH S7B protocol is employed to evaluate the potential of a studied drug and its metabolites to increase ventricular action potential duration (APD), thereby prolonging QT interval. This is accomplished by integrating the results from in vitro delayed rectifier potassium current (I_kr_) assays and in vivo QT assays. (i) In vitro assays: These assays use single cells or multicellular preparations to test a drug’s effect on a specific ion channel. Single cell preparations include heterologous expression systems in which human ion channel proteins are expressed in non-cardiac cells, as well as dissociated cardiomyocytes (e.g., guinea pig ventricular cardiomyocytes) that enable the assessment of the drug’s impact on action potential and ion currents. The effects of drugs are also tested on multicellular preparations such as Purkinje fibers, perfused myocardium and intact hearts. (ii) In vivo assays: These assays measure the effect of the tested drug on the QT interval using animal models, such as dogs, monkeys, rabbits and guinea pigs [23,24,25,26,27,28]. Since the QT interval is a representation of the average ventricular APD, QT interval prolongation by default results from increased APD. In turn, APD prolongation can result either from decreased outward (repolarizing) currents or increased inward (depolarizing) currents. Because QT interval is heart rate dependent, various formulas were proposed to generate a rate-corrected QT (QTc) to allow comparisons within individuals (e.g., upon drug exposure) or across populations [29]. The sensitivity of the in vivo assays is in the range of 64–82% and specificity of 75–88%; these low values imply that whereas a significant proportion of cardiotoxicity-free drugs will be identified as having cardiac ADRs and will be disapproved for marketing [28,30], other drugs will be cleared in the in vitro test as “safe”, albeit their cardiac ADRs will be discovered only post-marketing. For example, the low sensitivity (64%) and specificity (88%) of the hERG test (the rapid delayed rectifier potassium current, I_Kr_) are probably due to the fact that the biophysical characteristics of the potassium channels expressed in non-cardiac cells, are not identical to those of adult cardiomyocytes [31]. Unlike ICH S7B, the ICH E14 protocol is performed in vivo in humans, by measuring drug-induced QT prolongation or more precisely, the QTc interval, as a predictor of a pro-arrhythmic effect. There are a few limitations to using only the hERG/QTc assays: (i) Besides I_Kr_, other currents such as the L type Ca^2+^ current (I_Ca,L_) and fast inward Na^+^ current (I_Na_) contribute to ventricular repolarization [32,33]. (ii) QT interval prolongation alone is not ideal for predicting the clinical arrhythmia risk, and a prolonged QT interval may not always result in an arrhythmia. For example, Van Opstal et al. reported that chronic amiodarone does not evoke TdP, albeit reported QT prolongation in a canine model of acquired long-QT syndrome and chronic complete atrioventricular (AV) block [34]. In addition, clinical symptoms such as syncope can determine the clinical pro-arrhythmic risk. (iii) There are marked differences in the cardiac physiological and electrophysiological properties of humans versus experimental animal models, with different ion channel density and kinetics being species-dependent, thus diminishing the predictive value the latter [31,35,36]. Consequent to the abovementioned limitations of the ICH S7B and ICH E14 protocols for predicting cardiovascular toxicity, in 2013, the FDA proposed a new protocol (Comprehensive in vitro Proarrhythmia Assay, CiPA [37,38]) designed to be used in addition to the current protocols. The CiPA protocol has three preclinical and one clinical element: (i) determining the effects of the tested drug on multiple ion currents which contribute to ventricular repolarization (I_Kr_, I_Ks_ (the slow outward rectifier potassium current), I_Ca,L_, I_Na,L_ (the long-lasting sodium current), I_Na_, I_to_ (the transient outward potassium current) and I_K1_ (the inward rectifier potassium current) ); (ii) an in silico action potential (AP) model developed by the CiPA group based on the O’hara-Rudy human AP modified hERG model [39]; (iii) confirming the drug effects on action potentials and on ionic currents in human iPSC-CMs, to be described below at length; and (iv) a clinical element consisting of ECG evaluations from Phase 1 studies with the goal of detecting QT prolongation and changes in QT morphology that were not expected by the previous three preclinical stages. These unanticipated events may be caused by metabolites of the drug, changes due to protein binding of the drug or central nervous system effects [40].

### 2.5. Drug Development—Pharmacogenomics, Pharmacodynamics, Pharmacokinetics and Patient-Specific Effects

Patient-specific (adverse) reactions to a certain drug may arise due to interactions with genetic, non-genetic and environmental factors. A major field which addresses the influence of genetic variations on drug responses is Pharmacogenomics, which is the use of genomic and other “omic” information to individualize drug selection and drug use to avoid adverse drug reactions and to maximize drug efficacy [41]. For example, genetic variations in the expression and function of drug-metabolizing enzymes, can influence plasma drug concentrations, and thus cause ADRs. Pharmacogenomics is influenced by genetic variations as well as epigenetics, and may affect pharmacokinetic and pharmacodynamic properties. This can explain a patient specific response to a specific drug, along with ADRs; among the genetic variations, single-nucleotide polymorphisms (SNPs) are important players. SNP is a DNA sequence variation occurring when a single nucleotide adenine (A), thymine (T), cytosine (C) or guanine (G) in the genome (or other shared sequence) differs between members of a species or paired chromosomes in an individual. A common example is acquired long-QT syndrome (aLQTS) that is provoked by drug exposure, hypokalemia or bradycardia. Previous studies have shown that in 28% of cases rare pathogenic variants can be identified, and a QT interval polygenic risk score was developed based on the number of such variants in a specific patient. The higher is the risk score, the higher is the chance of QT prolongation with specific drug exposure [42,43]. An example of two drugs having different effects in specific populations are isosorbide dinitrate (BiDil) and hydralazine, both associated with a greater therapeutic effect in patients with a genotype TT of SNP rs5443 in the G-protein beta-3 subunit (GNB3). In this regards, the African American Heart Failure Trial (A-HeFT I) aimed to evaluate the use of BiDil to treat heart failure in African American patients, reported that the rs5443-TT genotype is found in ~50% of African Americans, whereas it is rare (~10%) in Caucasians [44]. The main findings were decreased mortality in African American patients treated with a fixed dose of BiDil compared to placebo; 10.2% mortality rate in the placebo group vs. 6.2% mortality rate in the BiDil group, representing 43% improvement in survival, whereas no difference was found in the Caucasians population. Hence, specific population’s (i.e., ethnicity of potential patients) SNPs and drug responses are important factors to be considered during a pharmacological development.

In addition to the inter-individual differences caused by polymorphisms, it is now evident that epigenetics further generates inter-individual differences in phenotype that may be of importance with respect to ADRs susceptibility. Epigenetics is usually defined as the study of mitotically heritable changes in gene expression that are not attributable to nucleic acid sequence alterations. In other words, epigenetics describes the mechanisms that enable cells to respond quickly to environmental changes and provide a link between genes and the environment. The main epigenetic phenomena in mammals are DNA methylation and histone modifications [45]. The study of mitochondrial epigenetics is developing in the last decade, and specifically the effect of mtDNA methylation in heart diseases. For example, Ikeda et al. proposed that mtDNA methylation impacts on transcription factor binding, such as that of Twinkle (TFAM), thereby influencing mitochondrial biogenesis and oxidative responses. TFAM over-expression reduces the hypertrophic response elicited by volume overload and reduces oxidative responses in the heart [46]. A potential target of epigenetic molecules is Histone DeACetylases (HDAC), which can be inhibited by several HDAC inhibitors (HDACi). For example, in a pressure-overload hypertrophy mouse model, trichostatin A and scriptaid (a broad-spectrum HDACi) were found to suppress the cardiac hypertrophy and fibrosis. Furthermore, the hypertrophy-associated switch of adult and fetal isoforms of myosin heavy chain expression was diminished [47]. In addition, these HDACi were shown to reduce myocardial infarct size and preserve systolic function in Langendorff-perfused hearts as well as in a mouse reperfusion model [48,49].

Due to the broad range of factors affecting ADRs incidence, a well-known target of modern medicine is “personalized medicine”; tailoring a specific treatment to a specific patient enables us to accomplish a high therapeutic efficacy, as well as minimize or even prevent ADRs. In summary, patient-specific drug responses include personal variations in pharmacogenomics, pharmacodynamics, pharmacokinetics, epigenetics and environmental factors. These variations are very difficult to discover in a small cohort of patients during the clinical phases of drug development [50,51], and therefore more specific guidelines are needed.

### 2.6. Drugs Adverse Effects—Different Mechanisms

This section focuses on three distinct mechanisms that affect the cardiac muscle directly: ion channels, intracellular Ca^2+^ handling and apoptosis.

#### 2.6.1. Ion Channels

Ventricular arrhythmias caused by APD prolongation are the most noticeable cardiotoxic side effect of drugs. Indeed, numerous clinical trials were terminated prematurely due to increased risk for life threatening ventricular arrhythmias, such as TdP [7,9,10,52,53,54]. Ventricular APD and QT interval prolongation can be caused by [7,9,10,52,53,54]: (i) decreased amplitude of the main repolarizing currents - I_Kr_, I_Ks_ and ultra-rapid (I_Kur_) potassium currents; and (ii) increased amplitude of the plateau-maintaining depolarizing currents, namely I_Na_ and I_Ca,L_ [55,56]. In turn, APD prolongation can result in generation of early afterdepolarizations (EADs) [57], which, if they exceed the electrical threshold, can trigger one or more arrhythmic action potentials, resulting in reentry, TdP, ventricular fibrillation and sudden cardiac death. As described below, drugs from different families cause APD and QT prolongation and present increased risk for life threatening cardiac arrhythmias. A “famous” example of post-marketing removal of drugs due to QT prolongation are the H-1-antihistamines (H1A), such as astemizole and terfenadine. After ~10 years of worldwide use, in the 1990s, these drugs were withdrawn from the market due to the risk of sudden cardiac death and ventricular tachyarrhythmia, associated with QT prolongation [52,53,54]. The proposed mechanism for the adverse effects of H1A drugs are: (i) effects on several ion currents such as I_Kr_, I_Ks_, I_Kur,_ I_Na_ and I_Ca,L_ [54,58]; and (ii) in neonatal rat ventricular cardiomyocytes, these drugs inhibit p21 (Cdc42/Rac)-activated kinase 1 (pak1) mRNA expression and function, resulting in altered expression profiles of genes involved in calcium transport/signaling [59].

#### 2.6.2. Intracellular Ca^2+^ Handling

Adverse effects of drugs on intracellular Ca^2+^ handling are associated with both electrical and contractile dysfunction, is another cardiotoxic mechanism. A key member of this notorious family is doxorubicin which impairs intracellular Ca^2+^ handling [60]. In 2010, Binah and co-workers [61] demonstrated that exposure of neonatal rat ventricular myocytes (NRVM) to doxorubicin (0.5 µM, 24 h) caused elevation of diastolic [Ca^2+^]_i_, slowing of [Ca^2+^]_i_ relaxation kinetics and decreased rates of myocyte contraction and relaxation. Further, doxorubicin reduced the protein expression of sarco/endoplasmic reticulum calcium (Ca^2+^ ATPase, Na^+^/Ca^2^ exchanger 1 and total connexin 43, as well as diminished gap junctional-mediated intercellular coupling and conduction velocity. Similarly, Burridge et al. demonstrated that doxorubicin treatment in iPSC-CMs increased the Ca^2+^ transient decay times with reduced Ca^2+^ transient amplitude and time to peak signal [62]. Similar adverse effects on intracellular Ca^2+^ handling are exerted by the Tyrosine Kinase Inhibitors (TKIs) nilotinib and vandetanib, which have a low cardiac safety index and were previously labeled with an FDA black box cardiotoxicity warning due to associated QT prolongation and arrhythmias [63]. QT prolongation is also induced by common antiarrhythmic drugs such as quinidine, dofetilide and sotalol, which cause TdP in 1–5% of treated subjects [29,64,65,66].

#### 2.6.3. Apoptosis

An important cause for severe cardiotoxicity is apoptosis (programmed cell death), which diminishes the number of functional cardiomyocytes, thereby causing depressed contractile function and heart failure. Several drug families, mainly chemotherapeutics, while having a desired cytotoxic effect responsible for the anti-cancer efficacy, can cause apoptosis, necrosis or both [67,68,69]. For example, anthracyclines represented by doxorubicin cause cardiomyocyte loss by inducing apoptosis, demonstrated in cultured cardiomyocytes including iPSC-CMs and experimental animals such as mice and rats [62,70,71,72,73,74,75,76,77,78,79,80,81,82]. Several groups showed that doxorubicin-induced apoptosis is mediated by the generation of reactive oxygen species (ROS) [75,76,83,84]. Another group of anti-cancer drugs which exert prominent cardiotoxicity are the Tyrosine Kinases Inhibitors (TKIs). In this regards, Doherty et al. showed that the TKIs crizotinib and nilotinib have cardiotoxic effects and increase superoxide generation, thus accounting for the drugs’ cardiotoxicity [85].

## 3. The Use of iPSC-CMs for Disease Modeling and Developing Novel Drugs for Heart Diseases

### 3.1. Induced Pluripotent Stem Cells, Pluripotency and Applications

As a brief background to this section, we provide a glimpse on the amazing technology of induced pluripotent stem cells (iPSCs). The world of biology and medical sciences underwent an incredible revolution with the remarkable breakthrough by Takahashi and Yamanaka in 2006 [86]. These prominent scientists discovered that by inserting four transcription factors (Oct3/4, Sox2, Klf4 and c-Myc) into fully-differentiated somatic cells, they are transformed/reprogrammed into iPSCs, which feature the two key properties of stem cells: they can divide indefinitely and are pluripotent. Once iPSCs are generated, they can give rise to practically any cell type, such as cardiomyocytes [87,88,89,90,91], neurons [92,93,94], pancreatic cells [95,96] and skeletal muscle cells [97,98]. The clinical implications of iPSCs are beyond imagination, since iPSCs can differentiate into a broad range of cell types which can be used for cell therapy, and tissue replacement and regeneration. Furthermore, as described in this review, iPSC-CMs are extensively used to investigate human cardiac physiology and pathophysiology, acquired and inherited cardiac diseases and drugs’ cardiotoxicity. Importantly, iPSCs have several advantages over human embryonic stem cells (ESC): iPSCs do not pose ethical issues (i.e., they are not derived from human embryos), their supply is unlimited and, for example, when iPSC-CMs are auto-transplanted, unlike ESC, immunosuppression is not required.

Prior to the introduction of iPSC-CMs, numerous research groups employed diverse in vitro models consisting of freshly isolated and cultured cardiomyocytes generated from a broad range of species such as dog, rabbit, guinea pig, rat and mouse [99,100,101,102,103,104,105,106]. While many studies using these models have produced valuable information respecting normal cardiac function and dysfunction under diverse experimental conditions, key fundamental features of these experimental models differ from those of the human heart. For example, exploring the mechanisms of Sino-Atrial Node (SAN) automaticity in pacemaker cells from rat and mouse which have heart rates of 330–480 [107] and 500–700 beats per minute (bpm) [108], respectively, are far from adequately representing the human heart which beats at 60–90 bpm. Further, in the mouse and rat, the ventricular action potential (AP) has a wedge-like appearance, thus lacking the plateau phase which characterizes human ventricular and Purkinje fiber APs. Hence, these and other dissimilarities between experimental animals and the human heart excitation–contraction-coupling machinery have highlighted the necessity for in vitro models of human cardiomyocytes.

#### 3.1.1. Promoting the Maturation of Immature iPSC-CMs

Despite the widespread use of iPSC-CMs for different purposes, soon after their introduction, it became clear that iPSC-CMs generated by different differentiation protocols exhibit diverse immaturity/maturity status compared to adult cardiomyocytes, and, hence, the urgent need to generate mature-like cardiomyocytes has emerged. Consequently, the goal of the maturation techniques employed by different groups was to accomplish iPSC-CM maturity, similar to that of adult cardiomyocytes, such that the derived cells display similar electrophysiological, Ca^2+^-handling and contractility performance, as well as autonomic and pharmacological responses [109]. Generating mature (adult-like) cardiomyocytes is important for the following reasons: (1) iPSC-CMs are the only currently available in vitro model of human cardiomyocytes. (2) Shortly following the breakthrough development of the iPSC technology and the generation of iPSC-CMs, major efforts have been directed (and still are) towards using these cells for cardiac muscle regeneration [110]. Hence, it is likely that mature (adult-like) cardiomyocytes are more suitable for regeneration than immature cardiomyocytes. (3) iPSC-CMs have become a preferable in vitro model for drug and toxicity screening [111]. Clearly, the closer (i.e., more mature) the iPSC-CMs properties are to those of the adult heart, the more reliable are the results of these tests. To meet the crucial need to generate adult-like cardiomyocytes, several maturation protocols have been developed. Among others, the procedures for causing iPSC-CMs maturation include manipulations of the culture conditions, the extracellular matrix [112,113], culture medium modifications [114,115,116,117,118,119], patterned seeding [120,121] and controlled microenvironment in a microfluidic system [122].

#### 3.1.2. Generating Isogenic iPSCs Using the Clustered Regularly Interspaced Short Palindromic Repeats (CRISPR)

Despite the prominent advantages of using patients’ iPSC-CM to model inherited cardiac diseases, a major barrier results from the need to discriminate between the effects of the causative mutation and the genetic background of these cells. Recent discoveries harnessing the adaptive immune system of prokaryotes to perform targeted genome editing, is having a transformative influence across the biological sciences. The discovery of Clustered Regularly Interspaced Short Palindromic Repeats (CRISPR) and CRISPR-associated (Cas) proteins, has expanded the applications of genetic research in thousands of laboratories across the globe and is redefining our approach to gene therapy [123,124,125,126,127]. Using a single non-sequence specific protein combined with a small guiding RNA molecule, this state-of-the-art approach enables modifications of genes with high efficiency and accuracy. By so doing, this technique enables the generation of isogenic controls or isogenic mutated cell lines in order to focus on the pathologies caused by a specific mutation. Briefly, in recent years, many groups including our own have used the CRISPR successfully to correct the mutation in the patients’ iPSCs, thereby generating isogenic control cell lines [90,128,129].

### 3.2. Acquired Heart Diseases

Of the numerus acquired heart diseases, this section addresses two important categories, which were modeled by means of iPSC-CMs: cardiotoxicity caused by anti-cancer drugs and cardiac hypertrophy.

#### 3.2.1. Cardiotoxicity Caused by Anti-Cancer Drugs

Contemporary anti-cancer treatments use a combination of radiotherapy and chemotherapy, including protein kinase inhibitors, and are associated with high risk of cardiotoxicity, which often limits their application [72,130,131,132]. Indeed, the common cardiovascular adverse side effects observed after chemotherapy, including myocarditis, pericarditis, myocardial ischemia, hypertension, QT interval prolongation, arrhythmias, thromboembolism and heart failure, constitute a major threat for short- and long-term life quality and overall outcome in cancer patients and survivors [133].

##### Cardiotoxicity Caused by Anthracyclines

Doxorubicin (DOX) is an anthracycline chemotherapeutic agent used for treating a wide range of malignancies such as acute leukemia [134], breast cancer [135] and non-Hodgkin lymphomas [136]. Doxorubicin can lead to cardiomyopathy associated with chronic heart failure as well as atrial and ventricular arrhythmias occurring within several days after treatment initiation [137,138]. In general, the extent and time course (acute versus chronic) of the cardiotoxicities are highly variable, patient- dependent and practically impossible to predict [139]. The cardiotoxicity caused by doxorubicin treatment varies from asymptomatic increase in left ventricular (LV) wall stress to arrhythmias, reduction in LV ejection fraction and highly symptomatic congestive heart failure, which might lead to a need for heart transplantation [140]. Although the cardiotoxicity of anti-cancer drugs is of clinical significance, the underlying mechanisms are not entirely clear. Anthracycline-induced cardiotoxicity was shown to induce cardiomyocyte apoptosis, mitochondrial dysfunction, disruption of intracellular calcium homeostasis and altered gene and protein expression [141]. Further, doxorubicin downregulates the myocardial expression of the transcription factor GATA4, a key regulator of sarcomeric proteins expression (e.g., myosin heavy chain and troponin), which is crucial for proper mammalian cardiac development [142,143,144]. Doxorubicin-induced cardiotoxicity (DIC) is characterized by the combination of massive reactive oxygen species (ROS) and nitric oxide synthase (NOS) [145,146] accumulation and lower levels of the antioxidant enzymes superoxide dismutase (SOD) and catalase (CAT) [145,146,147].

In accordance with the increased use of iPSC-CMs for investigating drug toxicity, several studies tested the cardiotoxic effects of doxorubicin, as described herein. However, despite the advantages of this model, its major limitation in DIC research is that it can only be used for assessing short-term (up to several days) cardiotoxicity, while some of the cardiotoxic effects in patients may be expressed during the long-term drug administration. Maillet and co-workers investigated DIC in human iPSC-CMs, and found that doxorubicin caused dose-dependent increases in apoptotic and necrotic cell death, reactive oxygen species production, mitochondrial dysfunction and increased intracellular calcium concentration. The group also characterized genome-wide changes in gene expression caused by doxorubicin using RNA-seq, as well as electrophysiological abnormalities using the multi-electrode array technology. This group showed that CRISPR-Cas9-mediated disruption of *TOP2B*, a gene implicated in DIC in mouse models, reduced the sensitivity of iPSC-CMs to doxorubicin-induced double stranded DNA breaks and cell death. These data establish a human cellular model of DIC that recapitulates many of the cardinal features of this adverse drug reaction and thus enables screening for protective agents against DIC as well as assessment of genetic variants involved in doxorubicin response [148]. In a study by Han and co-workers [149], the authors employed human iPSC-CMs to investigate the role of CircITCH in DIC and further decipher its potential mechanisms. They showed that CircITCH is a broad-spectrum tumor-suppressive circular RNA, and that its host gene ITCH is involved in DIC. Quantitative PCR and RNA in situ hybridization (ISH) revealed that CircITCH is downregulated in DOX-treated iPSC-CMs as well as in the autopsy specimens from cancer patients who suffered from DOX-induced cardiomyopathy. Cell death/viability assays, detection of cardiomyocyte necrosis markers, microelectrode array, and cardiomyocyte functional assays showed that CircITCH ameliorated DOX-induced cardiomyocyte injury and dysfunction. Based on their findings, the authors concluded that CircITCH represents a novel therapeutic target for DIC because it acts as a natural sponge of miR-330-5p, thereby upregulating SIRT6, survivin and SERCA2a to alleviate DOX-induced cardiomyocyte injury and dysfunction. Another important study was published in 2016 by Burridge and co-workers [62]; this group attempted to address the inability to predict which patients will be affected by DIC. They compared the cardiotoxic effects of doxorubicin in iPSC-CMs generated from women with breast cancer who experienced DIC (Group 1) to patients who did not experience DIC (Group 2). The main findings were that iPSC-CMs from Group 1 were more sensitive to DIC than iPSC-CMs from Group 2, demonstrating decreased cell viability, impaired mitochondrial and metabolic function, impaired calcium handling, decreased antioxidant pathway activity and increased reactive oxygen species production. Based on these results, the authors concluded that patient-specific iPSC-CMs can recapitulate the predilection to DIC of individual patients at the cellular level.

##### Cardiotoxicity Caused by Trastuzumab

Asides from anthracyclines, the use of targeted drugs inhibiting specific pathways critical for cancer progression, has also gained broad clinical use. Trastuzumab is a humanized monoclonal antibody used for treating breast and stomach cancer. Trastuzumab specifically recognizes cells expressing the HER2 receptors (only cancer cells express HER2 in significant quantities) [150] encoded by the *ErbB2* gene. HER2 is a transmembrane ligand-activated tyrosine kinase that controls cell proliferation, survival, differentiation and adhesion in response to neuregulin (and other ligands) binding. As a result of DNA mutations, HER2 can become overexpressed or continuously activated without the ligand, thereby causing cells over-proliferation [151]. Similar to doxorubicin, cardiotoxicity constitutes a significant side effect of trastuzumab treatment, thus limiting its use. Although the incidence rate of trastuzumab treatment discontinuation is mainly due to cardiotoxicity [152,153,154], the underlying pathological mechanisms are not entirely clear. In 2019, Kitani and co-workers [155] used human iPSC-CMs to investigate the mechanism underlying trastuzumab-induced cardiotoxicity, by determining the effects of trastuzumab on cardiomyocytes’ structural and functional properties. This group also generated iPSC-CMs from patients receiving trastuzumab, and investigated whether the patients’ phenotype could be recapitulated in vitro by using patient-specific iPSC-CMs. They found that clinically relevant doses of trastuzumab adversely affected the contractile and calcium-handling properties of iPSC-CMs without inducing cardiomyocyte death or sarcomeric disorganization. RNA-sequencing and subsequent functional analysis revealed mitochondrial dysfunction and altered cardiac energy metabolism pathway as primary causes of trastuzumab-induced cardiotoxicity. iPSC-CMs generated from patients who received trastuzumab and experienced severe cardiac dysfunction were more vulnerable to trastuzumab treatment than iPSC-CMs generated from patients who did not experience cardiac dysfunction following trastuzumab therapy. The authors concluded that alterations in cellular metabolism in cardiomyocytes is probably the main mechanism underlying cardiotoxicity caused by trastuzumab therapy, and therefore they proposed that targeting the impaired metabolism may be a promising therapeutic approach for trastuzumab-induced cardiotoxicity. Next, Kurokawa et al. [156] also employed iPSC-CMs to investigate the mechanisms underlying trastuzumab cardiotoxicity. Based on their finding this group concluded that iPSC-CMs can recapitulate the cardiotoxic effects of ErbB2 inhibition by trastuzumab and may be used to elucidate additional modes of toxicity of trastuzumab and related compounds.

##### Cardiotoxicity Caused by Tyrosine Kinase Inhibitors

Tyrosine kinase inhibitors (TKIs), which are small molecules interfering with the tyrosine kinases activity, were designed to disrupt cancer cell pathogenesis at specific biological points essential for tumor development and progression [157,158,159]. The introduction of TKIs to the anti-cancer arena has revolutionized the treatment of chronic myeloid leukemia, gastrointestinal stromal and renal cancer. The cardiotoxic effects of TKIs range from asymptomatic QT prolongation to a reduction in left ventricular ejection fraction (LVEF), symptomatic heart failure (HF), acute coronary syndromes, myocardial infarction (MI), hypertension and sudden death [160,161]. Sharma et al. [63] studied the cardiotoxic effects of 21 different TKIs in iPSC-CMs generated from patients receiving cancer treatment and healthy individuals. The highest magnitude of cardiotoxicity observed was induced by the VEGFR2/PDGFR-inhibiting TKIs. In a related study, Wang et al. [162] assessed iPSC-CMs response to four widely administered TKIs (sunitinib, sorafenib, lapatinib and erlotinib), using functional assays, microscopy, RNA sequencing and mass spectrometry. In general, the TKIs affected tyrosine kinase-mediated signal transduction and cardiac metabolism. The TKI sorafenib, particularly, disturbed the mitochondrial oxidative phosphorylation—the main source for ATP production. However, similar to tumors, as a compensation mechanism, the iPSC-CMs increased their rate of glycolysis. Accordingly, induction of glycolysis reduced sorafenib toxicity, presumably by providing an alternative source of ATP. In conclusion, this group found that TKI induce cardiotoxicity not only through inhibition of signal transduction but also through metabolic changes. Talbert et al. [163] investigated the use of iPSC-CMs for TKIs cardiotoxicity screening. They used the TKI ponatinib, which until recently was used to treat acute lymphocytic leukemia and chronic myeloid leukemia but was withdrawn from the market due to its cardiotoxicity effect. Ponatinib was reported to induce mitochondrial stress and elicit contraction abnormalities in iPSC-CMs.

#### 3.2.2. Cardiac Hypertrophy Induced by Hormones (AT-II) and Mechanical Stretch

Cardiac hypertrophy defined as an increase in cardiac mass, is a complex process compelled by simultaneous alterations in hemodynamics, mechanical stimuli and hormonal inputs, resulting in increased sarcomeric protein synthesis and assembly and cell size [164]. Hypertrophy occurs under “physiological” circumstances, for example during endurance exercise [165] or children’s growth. Cardiac hypertrophy also occurs in the course of pregnancy [166], where LV mass increases by ~30% [167] due to circulating hormone levels, which surge as the maternal cardiac output increases by 50% [168]. As the workload on the heart increases, the muscle tissue in the chamber wall further thickens, and sometimes the size of the chamber itself also increases. The enlarged heart muscle loses elasticity and eventually may fail to pump with as much force as needed, thus resulting in pathological hypertrophy. Hence, the abnormality of the hypertrophic cells may eventually impair their performance, rather than keep improving it, resulting in development of pathological hypertrophy [169]. The complex molecular mechanisms underlying the various forms of hypertrophy are beyond the scope of this review, and the interested readers are referred to the works of Wolf [170], Gallo et al. [171] Wehbe et al. [172] and Yan et al. [173].

One of the main hormones mediating hypertrophy is Angiotensin II (AT-II), which can cause hypertrophy independently of or in synergy with increased blood pressure. Further, AT-II has a key role in causing cardiac hypertrophy via several means involving direct actions on cardiomyocytes or the release of paracrine factors from other cell types such as endothelial cells and fibroblasts/myofibroblasts [174]. In endothelial cells, AT-II signal triggers release of Endothelin-1 (ET-1) that contributes to hypertrophy [175]. Similarly, AT-II signal triggers release of several factors (e.g., connective tissue growth factor (CTGF) and fibroblast growth factor–2 (FGF2) [176]) from fibroblasts, which augment hypertrophy. Mechanical stress was shown to be a promoting factor in releasing AT-II, as well as activation of its receptor (AT_1_R) even without the signal [177]; therefore, there is a strong connection between mechanical stress on the heart to its hypertrophic compensation mechanism.

Hypertrophy can be induced in vitro by mechanical stress, hormones or their combination. Until recent years, the most common cell model used was neonatal rat ventricular myocytes (NRVM). Archer et al. [178] demonstrated expression of hypertrophic markers in NRVM after a 15-min pulse of ET-1. Further, Miyata et al. [179] showed that mechanical stretch activates the cardiac renin-angiotensin system (RAS) in an autocrine and paracrine fashion, which acts as an initial mediator of the stretch-induced hypertrophic growth in NRVM. In recent years, several groups utilized a variety of experimental means to induced hypertrophy in iPSC-CMs. Tanaka et al. [180] studied the ability of ET-1, AT-II, insulin-like growth factor 1 and phenylephrine (PE) to induce hypertrophy in iPSC-CMs. The main findings were that ET-1 treatment increased cell area, myofibrils disarray and increased NFATc4 translocation to the nucleus. Further, endothelin receptor type A blocker (ETA-b) distinctly prevented ET-1 induced hypertrophic changes. Next, Ruan et al. [181] demonstrated in iPSC-CMs that static stress (SS) and SS combined with electric pacing (SE) increase cell size (measured by immunostaining of myosin heavy chain-7 (MYH7) antibody) by ~50%. SS was achieved by maintaining iPSC-CM constructs at a fixed static length for two weeks, whereas SE was achieved by one week of SS followed by a second week of SE at 2 Hz. In another study, Rupert et al. [182] investigated the effects of PE (72 h, 2 µM PE) on the 2D and 3D aspects of iPSC-CMs, and found that while PE increased cell size by 70%, cell volume was unaffected, indicating that hypertrophic changes in iPSC-CMs were due to PE effects on the cells’ 2D aspect.

### 3.3. Inherited Heart Diseases

As demonstrated by numerous studies, patients’ specific iPSC-CMs constitute a superb experimental model to explore a broad range of inherited cardiac diseases, such as cardiac arrhythmias. As nicely stated by Gintant and Traebert [183], “the use of specific patient-derived hiPSC-CMs could guide personalized pharmacologic assessments of pharmacologic therapies (for example, safety of antineoplastic agents with narrow therapeutic margins) as well as complement personalized therapeutic approaches for selecting the most efficacious drug or drug combinations (the later studies also involving combination in vitro drug studies before proceeding to humans)”.

#### 3.3.1. Structural Mutations

##### Hypertrophic Cardiomyopathy (HCM)

HCM, the most frequently occurring cardiomyopathy with a prevalence of 0.02–0.23% in different adult populations [184], is characterized by asymmetric or concentric LV wall thickening, in the absence of another causative factors [185,186,187], with major outcomes being contractile dysfunction and potentially fatal arrhythmias [188,189,190,191,192]. The severity of the disease varies from a lifelong asymptomatic course to early disease onset associated with ventricular arrhythmias, severe heart failure and sudden cardiac death (SCD) in young adults and apparently healthy athletes [193]. Thus far, over 400 familial HCM-causing mutations have been identified [194]; in 60–70% of these cases, components of the sarcomere whose integrity is essential for proper heart contractility, are affected [185,195,196]. Thus, sarcomere defects provide a logical explanation for the development of the disease and myocardial disarray.

##### Mutations in the *MYH7* Gene

The *MYH7* gene [197] encodes the myosin heavy chain beta (MHC-β) isoform expressed primarily in the heart and skeletal muscles, and plays a major role in contraction. Whereas MHC-β is the predominant protein in the thick filament the human heart [198,199,200], MHC-α is the major isoform in the mice and rats [201,202,203]. Over 60 different mutations in the *MYH7* gene accounted for approximately 30% of HCM cases [204]. Importantly, besides the few studies/genes presented here, there are many more prominent reports not addressed due to space constraints. For a more thorough review, see for example an excellent review by Eschenhagen and Carrier [205], which lists many studies (and genes) of both HCM and DCM not mentioned here. Two comprehensive studies of HCM caused by different *MYH7* mutations were performed in recent years, using iPSC-CMs models [206,207]. The first included 10 family members, five of which carried the well-known familial missense mutation R663H on exon 18 of the *MYH7* gene, while the other five did not present the mutation. iPSC-CMs from the patients carrying the mutation exhibited structural abnormalities such as cellular enlargement and multinucleation, increased myofibril content and high percentage of cells with disorganized sarcomeres. Respecting the functional abnormalities, the mutated iPSC-CMs demonstrated increased hypertrophy-related protein (e.g., ANF) levels and elevation of β-myosin/α-myosin ratio, as well as HCM-related genes (e.g., *GATA4* and *TNNT2*), increased intracellular Ca^2+^ concentration ([Ca^2+^]_i_) and arrhythmias on a single cellular level. The group also found that the I_Na_ blockers lidocaine, mexiletine and ranolazine, and the I_Ca,L_ blockers verapamil, diltiazem and nifedipine attenuated the adverse changes in the HCM iPSC-CMs. In contrast, the β-blockers propranolol and metoprolol, and the K^+^ channel blockers amiodarone, sotalol and dofetilide were ineffective [206]. The second study [207] was performed on iPSC-CMs derived from a 37-year-old female patient carrying the single missense mutation R442G in the *MYH7* gene. Morphologically, the mutated iPSC-CMs exhibited enlarged surface area and disrupted sarcomere organizations, in agreement with the known HCM phenotype. Functionally, electrophysiological abnormalities such as prolonged action potential (AP) duration and changes in AP shape were found in the mutated iPSC-CMs, compared to control. In addition, compared to control, HCM iPSC-CMs exhibited elevated resting [Ca^2+^]_i_ as well as a lower caffeine-induced increase in Ca^2+^ transient, indicating decreased SR-Ca^2+^ content. Additionally, in mutated iPSC-CMs compared to control, I_Ca,L_, I_Na_ and outward K^+^ currents were larger. Further, in agreement with Han and co-workers [207], this group found that verapamil and the histone deacetylase inhibitor trichostatin A (TSA) attenuated the various HCM-related abnormalities in the mutated cells.

##### Mutations in *MYBPC3* Gene

The *MYBPC3* gene encodes the cardiac myosin-binding protein C, which is crucial for sarcomere organization and proper cardiac function. Mutations in *MYBPC3* are another common cause of HCM, being responsible for an estimated 35% of cases [208]. Most of *MYBPC3* mutations result in premature termination codons, causing protein deficiency [209]. A recent study by Seeger and colleagues used iPSC-CMs from three HCM patients: (i) a patient carrying c. 2827 C > T; p.R943x mutation in the *MYBPC3* gene resulting in a PTC in exon 27; (ii) an unrelated patient carrying the same mutation; and (iii) a patient carrying the p.R1073P_Fsx4 mutation in the *MYBPC3* gene. This study showed that mutated iPSC-CMs exhibit elevated diastolic [Ca^2+^]_i_ and prolonged relaxation kinetics, compared to isogenic control cells. In addition, the expression of *ATP2A2* encoding for cardiac SR Ca^2+^ ATPase was downregulated in the mutated cells. Furthermore, the RNA-Seq analysis revealed a gene signature of the nonsense-mediated decay (NMD) pathway in the mutated iPSC-CMs compared to isogenic control. A small-interfering RNA approach was used to inhibit the key component of the NMD pathway—*UPF1*. In the mutated cells, *UPF1* inhibition led to the normalization of the gene profile expression, as well as to the significant improvement in the mutated iPSC-CMs Ca^2+^ handling [128].

##### Dilated Cardiomyopathy (DCM)

DCM can be caused by several factors, including genetic diseases, infections, alcohol and cocaine usage, toxins and pregnancy complications [210,211]. Familial cases were identified in 30–50% of diagnosed DCM patients [212,213,214,215]. Genetic mutations were identified in over 40 different genes, with inheritance pattern of autosomal dominant, autosomal recessive, X-linked and mitochondrial fashion [195,216,217,218,219].

##### Duchenne Muscular Dystrophy (DMD)

**Figure 3:**
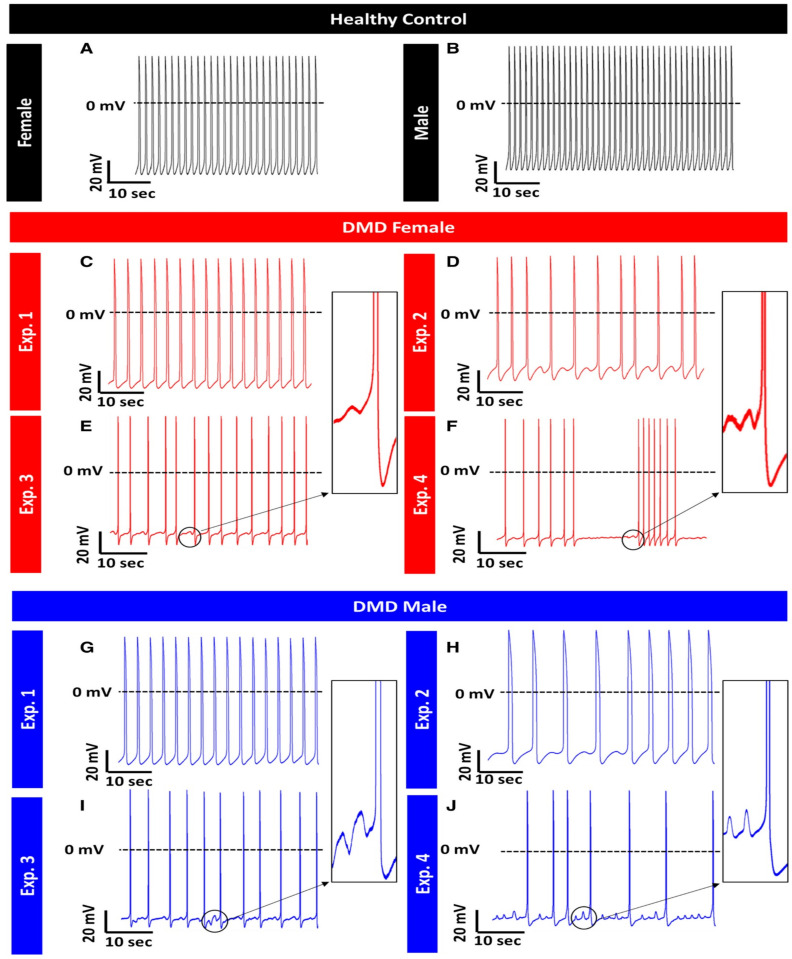
Representative spontaneous action potential recordings from control and DMD iPSC-CMs: (**A**,**B**) control iPSC-CMs do not manifest arrhythmias during spontaneous activity; (**C**–**F**) DMD female iPSC-CMs display arrhythmogenic firing pattern including delayed afterdepolarizations (DADs) and oscillatory prepotentials (OPPs) in 52% of spontaneous recordings (in (**D**–**F**)); and (**G**–**J**) DMD male iPSC-CMs display arrhythmogenic firing pattern including DADs and OPPs in 17% of spontaneous recordings (in (**H**–**J**)). Control, *n* = 25; DMD female, *n* = 21; DMD male, *n* = 23. Adopted from [91].

DMD is an X-linked progressive muscle degenerative disease caused by mutations in the *dystrophin* gene encoding the dystrophin protein [220,221]. In skeletal and cardiac muscle cells, dystrophin provides mechanical stability essential for the contracting myocytes and anchors the cellular cytoskeleton to the extracellular matrix (ECM) via the transmembrane dystrophin–glycoprotein complex (DGC), which links directly to extracellular laminin [222,223,224]. Absence of dystrophin leads to loss of myocyte anchoring to the surrounding ECM, and over time microlesions are formed in the contracting sarcolemma [225]. In addition, loss of structural integrity results in depolarization of the membrane resting potential and [Ca^2+^]_i_ overload [226,227]. Cardiac involvement in DMD is exhibited in half of the male patients by the age of 18, while the manifestation of skeletal muscle damage is observed in early childhood, before the age of five. DMD DCM is associated with heart failure accompanied by various atrial and ventricular arrhythmias [228,229,230,231]. Studies of DMD patients reported autonomic nervous system dysfunction leading to higher heart rate and altered heart rate variability (HRV) parameters. Overall, DMD patients suffer from increased sympathetic activity, which along with decreased contractile function is consistent with heart failure resulting from DCM [232,233,234,235]. A study performed by Lin and colleagues in DMD iPSC-CMs displayed increased diastolic [Ca^2+^]_i_, mitochondrial damage and enhanced cell apoptosis due to an activated mitochondria-mediated signaling network: from damaged mitochondria→DIABLO→XIAP→CASP3 cleavage→apoptosis. Further, the membrane sealant Poloxamer 188 attenuated the increase in [Ca^2+^]_i_ and suppressed apoptosis in the mutated cells by repressing caspase-3 activation [236]. In a recent study from Binah’s lab [91], we investigated iPSC-CMs from one male and one female DMD patients. Although DMD is an X-linked disorder, in some cases female patients exhibit clinical abnormalities [237]. In our study both DMD male and female patients’ iPSC-CMs presented low spontaneous firing rate, prominent arrhythmias (Figure 3) and prolonged action potential duration. iPSC-CMs from the male patient exhibited decreased amplitude of the key pacemaker current *I_f_*, while iPSC-CMs from the female DMD patient presented with increased beat rate variability. In addition, both female- and male-derived iPSC-CMs showed increased I_Ca,L_ density [91].

#### 3.3.2. Channelopathies

**Figure 4:**
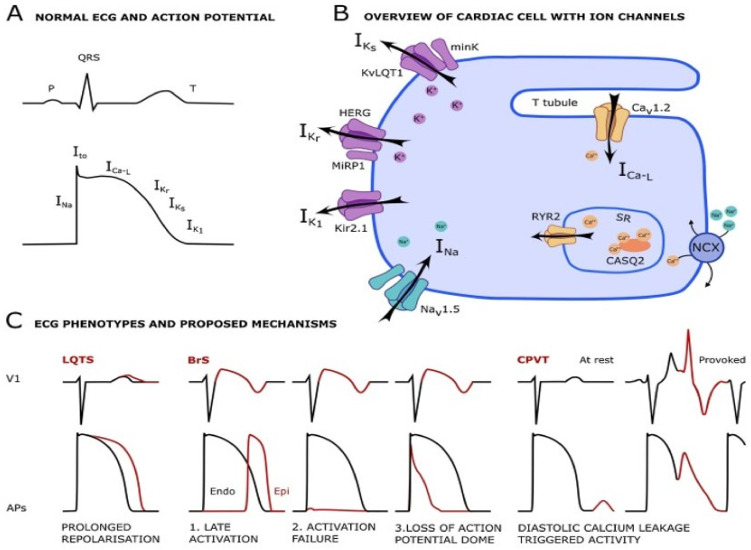
(**A**) The cellular action potential driving the heart cycle is shaped by a series of depolarizing and repolarizing ion currents (I). The major depolarizing currents in ventricular cardiomyocytes are sodium (I_Na_) and L-type calcium currents (I_Ca-L_). The major repolarizing currents are potassium currents (transient outward current I_to_, rapid delayed rectifier current I_Kr_ and slow delayed rectifier current I_Ks_). I_K1_ is an inward rectifier current maintaining resting membrane potential and controlling cellular excitability. (**B**) The ion channels and related proteins responsible for depolarizing (Na_V_1.5 and Ca_V_1.2) and repolarizing (KVLQT1/minK, HERG/MiRP1 and Kir2.1) currents are found in the cell membrane, either on the cell surface or in the transverse tubules (T tubules). The sodium/calcium exchanger (NCX1) contributes to the depolarizing current via changing 3 sodium ions (Na^+^) for 1 calcium ion (Ca^2+^), resulting in a net positive inward current. Calcium handling and control during cardiomyocyte contraction and relaxation is mediated by the process of calcium-induced calcium release from the sarcoplasmic reticulum (SR), where calcium is bound by calsequestrin (CASQ2) and released into the cytosol by the ryanodine-receptor (RYR2) channel. (**C**) Suggested mechanisms of the Long QT Syndrome (LQTS), Brugada and Catecholaminergic Polymorphic Ventricular Tachycardia (CPVT) ECG patterns, as seen in the first precordial lead (V1). APs: Ventricular action potentials. LQTS: Reduced repolarizing currents (I_Ks_ in LQT1 and I_Kr_ in LQT2) or increased depolarizing currents (I_Na_ in LQT3) result in a prolonged repolarization and a prolonged QT interval on the ECG. BrS: Three alternative pathophysiological mechanisms underlying the type 1 Brugada pattern have been proposed: (1) late activation of the right ventricle causes ST-segment elevation and repolarization of the same myocardium causes the negative T-wave; (2) excitation failure at the right ventricular subepicardium causes ST-segment elevation and moderate activation delay at neighboring sites causes the negative T-wave; and (3) loss of the action potential dome at the right ventricular subepicardium but not the subendocardium; i.e., transmural dispersion in action potential duration. CPVT: Resting ECG features in CPVT are typically normal. Dysfunction of the sarcoplasmatic reticulum calcium release channel or calcium storage causes leakage of calcium in diastole, and increasing intracellular calcium concentrations causes delayed afterdepolarizations and extrasystolic action potentials, which may trigger polymorphic VT [246].

Clearly, the field of “Channelopathies” has expanded extensively in recent years, and it can only be addressed briefly in the article. A schematic overview of the major ion channels involved in channelopathies-related arrhythmias is presented in Figure 4. Recent excellent reviews on channelopathies were published by Abriel and Zaklyazminskaya [238], Waddel-Smith et al. [239], Fernández-Falgueras et al. [240], Garcia-Elias et al. [241], Kline and Costantini [242], Wu et al. [243], Chahal et al. [244] and Torrente et al. [245].

##### Long QT Syndrome

Long-QT syndrome (LQTS) is a congenital disorder of the cardiac electrical activity, which predisposes the patients to sudden, life-threatening ventricular arrhythmias and is characterized by QT prolongation [239,247] (Figure 3). The normal range of QT intervals in ECG is 370–440 ms and varies according to the heart rate. Values of < 340 ms and > 460 ms are considered pathologic and typify short-QT syndrome (SQTS) and LQTS, respectively. In LQTS, ECG generally reveals QT interval repeatedly longer than 470 ms in women and 450 ms in men in the absence of other known pathologies affecting the QT pattern. Borderline cases of 340–370 and 440–460 ms represent milder types of QT interval abnormalities or variations in healthy individuals. The prevalence of LQTS ranges from 1:2000 to 1:10,000 due to variations in penetrance and geographic differences, with a minor female predominance [248,249]. Despite the similarity in genotypic sex-distribution, adult women are present with more prolonged QT intervals, and hence may undergo clinical diagnosis earlier compared to men. The phenotypic expression of LQTS is recurrent syncope during stress or rest, depending on the LQTS type. These arrhythmic episodes are due to Torsades de Pointes (TdP) which might terminate spontaneously without external intervention or deteriorate to ventricular fibrillation (VF), causing sudden cardiac death (SCD). The rate and severity of the clinical symptoms such as age of onset and prevalence of syncope, arrhythmias and SCD can broadly vary even among individuals from the same family. Respecting the underlying mechanisms, LQTS falls under the category of channelopathies and is caused by mutations in genes which encode for cardiac ion channels. Until 2019, 17 subtypes of congenital LQTS have been identified, each resulting from mutations in different genes [246]. The most prevalent subtypes, LQT1 and LQT2, account for the majority of congenital LQTS and are associated with mutations in the *KCNQ1* and *KCNH2* genes, respectively, which encode for proteins involved in I_Ks_ and I_Kr_ currents.

**Figure 5:**
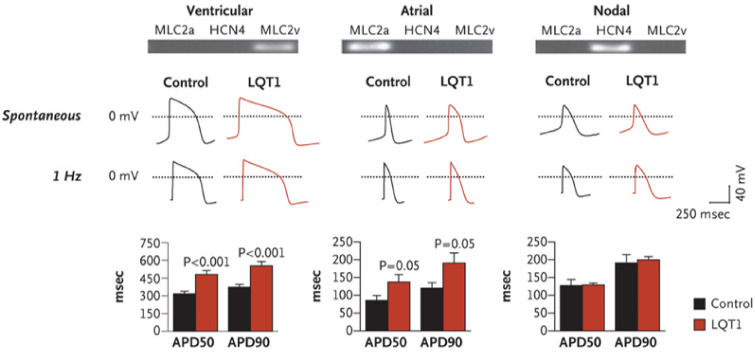
(**Top**) The results of single-cell RT-PCR from three representative patched cells with respect to the expression of specific myocyte markers: MLC2v, MLC2a and hyperpolarization-activated, cyclic nucleotide-gated channel 4 (HCN4). (**Middle**) Examples of typical spontaneous and paced (at 1 Hz) “ventricular”, “atrial” and “nodal” action potentials recorded in cardiomyocytes derived from induced pluripotent stem cells from a control subject and a patient with LQT1. (**Bottom**) The bar graphs represent the averaged action-potential duration at 50% repolarization (APD50) and at 90% repolarization (APD90) for each of the three myocyte subtypes at a 1-Hz stimulation rate. Between 4 and 40 cells from six different induced pluripotent stem-cell clones (three clones from each person) were analyzed per group. P values are for the comparison between LQT1 myocytes and control myocytes, with the use of one-way analysis of variance [87].

Soon after iPSC-CMs became acceptable/attractive for modeling inherited heart diseases, several groups employed iPSC-CMs generated from LQTS patients carrying different disease-causing mutations such as *KCNQ1* and *KCNH2*. Moretti et al. [87] generated iPSC-CMs from members of a family affected by LQTS type 1 caused by missense mutation in the *KCNQ1* gene. As shown in Figure 5, individual cells showed a “ventricular”, “atrial” or “nodal” phenotype, as evidenced by the expression of cell-type–specific markers and as seen in recordings of the action potentials in single cells. Action potential duration (APD) was markedly prolonged in “ventricular” and “atrial” in the mutated cells compared to cells from control subjects. Further characterization of the role of the R190Q–KCNQ1 mutation in the pathogenesis of long-QT syndrome type 1 revealed a dominant negative trafficking defect associated with a 70–80% reduction in I_Ks_ amplitude and altered channel activation and deactivation properties. Further, the mutated cells had increased susceptibility to catecholamine-induced tachyarrhythmia and β-blockade attenuated this phenotype. In a similar study, Itzhaki et al. [88] investigated iPSC-CMs generated from type-2 LQTS patient carrying the A614V missense mutation in the *KCNH2* gene; they found that APD prolongation in the mutated cells resulted from a reduction in *I*_Kr_. Further, LQTS-cardiomyocytes showed early-afterdepolarizations (EADs) and triggered arrhythmias. Because malignant arrhythmias in LQTS patients are often precipitated by drugs that block *I*_Kr_, this group assessed the effects of the specific *I*_Kr_ blocker E-4031. They found that E-4031 prolonged (by 47%) APD in the mutated iPSC-CMs and increased arrhythmogenesis manifested by increased EADs number and complexity. In contrast, the Ca^2+^-channel blocker nifedipine shortened APD at 90% repolarization (APD_90_) by 57% and eliminated all EADs and triggered beats. Using a different approach to attenuate arrhythmogenesis, this group showed that pinacidil (a K_ATP_-channel opener) shortened APD_90_ and abolished EADs and triggered arrhythmias. Finally, this group found that, while the late Na^+^-channel blocker, ranolazine [250], did not affect ADP_90_, it exerted a pronounced anti-arrhythmic effect. Rather than generating mutated iPSC-CMs from LQTS patients, the ion channel genes *KCNQ1* and *KCNH2* with dominant negative mutations causing long-QT syndrome types 1 and 2, respectively, were stably integrated into a safe harbor AAVS1 locus using zinc finger nuclease technology [251]. This excellent study showed that the edited iPSC-CMs featured characteristic long-QT syndrome phenotype and APD prolongation, compared with the unedited control cells. Further, nifedipine (L-type calcium channel blocker) or pinacidil shortened APD of iPSC-CMs, confirming the validity of isogenic iPSC lines for drug testing in the future. Patch-clamp recording demonstrated significantly prolonged APD compared to isogenic control cell lines. Furthermore, EADs were present in both patient-specific and edited iPSC-CMs but not in the unedited cell lines. Hence, the generated ZFN-edited LQTS iPSC lines share similar properties with mutation-matched iPSC-CM, and thus recapitulate LQTS phenotype faithfully. Next, this group found that nifedipine shortened APD in both patient-specific iPSC-CMs and the ZFN-edited iPSC-CMs. Calcium transient recordings also indicated a similar response to nifedipine; compared to control, both edited and patient-derived LQTS cardiomyocytes had prolonged calcium transient duration and marked shortening following nifedipine treatment. Collectively, these data suggest that genome-edited iPSC can serve as a model for drug screening.

##### Timothy Syndrome—LQTS Type 8

Timothy syndrome is a multisystem disorder caused by gain-of-function mutations in the L-type calcium channel Ca_V_1.2 encoded by *CACN1C* gene, leading to LQTS type 8, and additional impairments such as webbing of fingers and toes, immune deficiency, intermittent hypoglycemia, cognitive abnormalities and autism [252]. In 2011, Yazawa and co-workers [253] generated iPSC-CMs carrying a mutation causing a single amino acid substitution in exon 8a of *CACNA1C* (the gene encoding Ca_V_1.2 in humans), causing Timothy syndrome. Electrophysiological recordings and Ca^2+^ imaging studies of these cells revealed irregular contractions, excessive Ca^2+^ influx, prolonged APDs, irregular electrical activity and abnormal Ca^2+^ transients in ventricular-like cells. Further, roscovitine, a cyclin-dependent kinase inhibitor which increased the voltage-dependent inactivation of Ca_V_1.2 [254,255,256], restored the electrical and Ca^2+^ signaling properties of cardiomyocytes from Timothy syndrome patients.

##### Short QT Syndrome

Short QT syndrome (SQTS) is a rare inherited arrhythmogenic disorder characterized by short QT interval (QTc ≤ 340 ms), leading to syncope, life-threatening tachyarrhythmias and SCD [257,258], with QTc ≤ 330 ms, significantly shorter than in healthy equivalent. Due to ion channels malfunction, the cardiac repolarization period is accelerated and shortens the refractory period, which in turn may cause severe ventricular arrhythmias. The clinical manifestations range from asymptomatic patients to dizziness, palpitations, atrial fibrillation (AF), tachyarrhythmias, syncope and SCD. The age of symptoms onset varies across different decades of life, albeit SCD is occasionally the first symptom of the cardiac condition, such as in LQTS. LQTS can present in infancy and is a cause of SIDS [259]. Since a relatively scarce number of cases exists worldwide, it is difficult to assess the actual prevalence properly; recent reports suggest a prevalence of 0.05% in pediatric population and 0.02–1% among adults [260]. SQTS is a genetically heterogeneous disease with an autosomal-dominant inheritance, involving multiple genes identified so far to be linked with the syndrome. The most abundant SQTS subtypes are associated with gain-of-function of mutated potassium channels, especially in the *KCNH2* gene (SQTS type1) and/or loss-of-function mutations in genes encoding for various subunits of cardiac L-type Ca^2+^ channel [261]. Hence, a decrease in inward depolarization currents and/or an increase in outward repolarization currents shorten APD, abbreviate the QT interval and predispose the patient to reentrant arrhythmogenic mechanisms that may lead to VT and VF [262]. It is important to note the low yield (15%) of genetic screening for the syndrome, which further advocates its heterogenicity.

To investigate SQTS, several groups recently generated iPSC-CMs from SQTS patients. Pharmacological studies in SQTS type 1 patients showed that, of the antiarrhythmic drugs quinidine, nifekalant, disopramide, flecainide and sotalol, only quinidine was effective [257,263,264,265]. El-Battrawy and co-workers [266] generated iPSC-CMs from a SQTS type 1 patient carrying the N588K mutation in the *KCNH2* gene. Electrophysiological experiments demonstrated shortened ADP, increased I_Kr_ density, aberrant Ca^2+^ transients and increased arrhythmic events compared to control cell lines. Further, the SQTS type 1 iPSC-CMs were treated with the antiarrhythmic drugs quinidine, sotalol and metoprolol (used to treat patients with mutated I_Kr_); while none of the drugs affected action potential amplitude and resting potential, quinidine reduced V_max_, prolonged APD and diminished carbachol-induced arrhythmias. These data are consistent with previous in vitro and in vivo studies and illustrate the ability of patient-specific iPSC-CMs to recapitulate the disease phenotype in vitro. In another study, Shinnawi et al. [129] modeled SQTS type 1 using iPSC-CMs generated from symptomatic SQTS patient carrying the N588K mutation in the *KCNH2* gene. SQTS type 1 iPSC-CMs exhibited shortened APD and refractory period and increased I_Kr_ density due to attenuated inactivation. Pharmacological testing demonstrated that both quinidine and disopyramide rescued the disease phenotype and suppressed the arrhythmia by prolonging APD. Treatment with sotalol did not affect SQTS iPSC-CMs, as it failed to prolong APD, thereby being ineffective in preventing arrhythmogenic events. These results are concordant with clinical finding in N588K-SQTS1 patients in which sotalol did not improve the disease phenotype. Finally, Zhao and co-workers [267] used iPSC-CMs from a SQTS type 1 patient, aiming at searching for potentially effective drugs. Ivabradine, mexiletine and ajmaline but not flecainide, ranolazine or amiodarone prolonged APD. Ivabradine, ajmaline and mexiletine inhibited the KCNH2 channel currents, probably underlying their APD-prolonging effects. Under proarrhythmic epinephrine stimulation in spontaneously beating SQTS type 1 iPSC-CMs, ivabradine, mexiletine and ajmaline but not flecainide reduced the epinephrine-induced arrhythmic events. These authors concluded that ivabradine, ajmaline and mexiletine should be considered as candidate drugs for preventing tachyarrhythmias in SQTS type 1 patients.

##### Brugada Syndrome

Brugada syndrome (BrS) is a rare, inherited autosomal dominant cardiac disease, leading to life-threatening ventricular arrhythmias and high risk of SCD in the absence of structural cardiac anomaly. BrS was first described in 1992 as a distinct syndrome characterized by a unique ECG pattern consisting of coved type ST elevation followed by T wave inversion in right precordial leads, leading to high risk SCD [268]. Current data suggest that BrS is responsible for 5–40% of all sudden deaths in patients with structurally normal heart and a major cause of death in men < 40 years living in endemic areas [247,269,270,271]. The prevalence of the BrS ECG pattern varies greatly between populations, with higher prevalence in Asian populations (0.15–0.27%) and lower in Western countries (0–0.1%). The prevalence across genders is also disproportional, with disease expression higher in male [272]. BrS is usually manifested during adulthood, with SCD as the first clinical event in some individuals [273]. Others will most likely suffer from arrhythmic events such as paroxysmal supraventricular tachycardia (PSVT), atrial fibrillation (AF) and ventricular tachycardia. The triggers mainly include fever, electrolyte imbalance, alcohol, cocaine, large food intake and a variety of medications [274]. In up to 20–40% of genetically diagnosed affected individuals, a direct mutation in *SCN5A* gene can be linked [275], the first mutation that has been identified and the most common cause of BrS. The *SCN5A* gene encodes for the α subunit of the cardiac voltage-gated sodium channel Na_V_ 1.5 responsible for I_Na_. The resulting loss-of-function mutation, which decreases I_Na_, accounts for 25–30% of the genotyped BrS [276]. The remaining BrS genetically diagnosed cases are linked with a variety of mutations, mainly resulting in reduced I_Na_ or I_Ca,L_. Other than sodium channels, pathogenic variants in several subunits of potassium channels, such as potassium voltage-gated and inward rectifier channels, have been reported to cause BrS, though their implication in BrS has been disputed and currently only *SCN5A* has been found to be associated with BrS [277,278]. Li et al. [279] investigated the pathophysiological phenotype of two BrS patient-derived iPSC-CMs carrying a heterozygous nonsense mutation in the *SCN5A* gene. BrS iPSC-CMs presented a 50% decrease in peak I_Na_ density, a ~70% reduction of Na_V_1.5 expression and impaired localization of Na_V_1.5 and connexin 43 at the cell surface, compared to control iPSC-CMs. BrS iPSC-CMs also display decreased action potential upstroke velocity and conduction slowing. In addition, the transient outward potassium current (I_to_,) was enhanced. These results demonstrate the ability of Brs patient-derived iPSC-CMs to recapitulate the loss-of-function of Na_V_1.5. To test potential pharmacological therapies, the authors tested two clinically used phosphodiesterase (PDE) inhibitors cilostazol and milrinone to suppress arrhythmias [280]. Treatment with 10 µM cilostazol at +60 mV pulse stimulation for 1.5 h resulted in a 51% decrease in I_to_ density in BrS1 iPSC-CMs and 35.4% in BrS2 iPSC-CMs, while control cells were only slightly and non-significantly affected (14.4%). Analogous treatment with 2.5 µM Milrinone produced similar pattern of notable I_to_ current reduction in BrS iPSC-CMs (36.3% and 40%, respectively) with a non-significant reduction of 10.9% in control cells. In addition, both cilostazol and milrinone reduced the arrhythmia incidence from 40.5% arrhythmias-presenting cells in untreated cultures to 21% and 17% in BrS iPSC-CMs, respectively. These data strengthen the therapeutic efficacy of cilostazol and milrinone in antiarrhythmic drug management of BrS patients.

#### 3.3.3. Inherited Arrhythmias Resulting from Non-Ion Channel Mutations

Under this category, we focus on catecholaminergic polymorphic ventricular tachycardia (CPVT). This disease is an arrhythmic cardiac disorder, characterized by episodic syncope or SCD triggered by exercise or an emotional response, in the absence of structural heart disease or QT prolongation [247,281,282]. While resting ECG is typically normal, the triggers provoke sudden polymorphic or bidirectional ventricular tachyarrhythmias. These arrhythmias may either resolve spontaneously or deteriorate to ventricular fibrillation and cardiac arrest. The prevalence of the disease is estimated at 1:10,000 and usually presents at a mean age of 10 [249]. Specifically, there is a bimodal presentation with RYR2, positive CPVT presenting earlier in life (with males having more risk to develop events) and non-genotyped CPVT presenting at an older age and more in women [283]. If untreated, before the age of 30 years CPVT causes high mortality rate of 30% in affected individuals until the age of 20 years. Two genetic subtypes were identified to underlie CPVT: CPVT1, an autosomal dominant form accounting for ~65% of all genotyped CPVT cases, is associated with mutations in the gene encoding the cardiac ryanodine receptor (RYR2), the calcium-releasing channel at the sarcoplasmatic reticulum (SR). CPVT2 is autosomal recessive and less common, caused by mutations in the calcium-binding protein calsequestrin (CASQ2). Additional rare cases have been associated with several other calcium-handling genes such as CALM1 and TRDN, encoding for calmodulin and triadin, respectively.

In a report from Binah’s lab [89], we were the first to generate iPSC-CMs from a CPVT2 patient carrying the missense mutation D307H in the cardiac calsequestrin gene *CASQ2*. The major findings were that the β-adrenergic agonist isoproterenol caused in CPVT2 iPSCs-CMs (but not in the control cardiomyocytes) DADs, oscillatory arrhythmic pre-potentials, after-contractions and diastolic [Ca^2+^]_i_ rise. Electron microscopy analysis revealed that, compared with control iPSC-CMs, CPVT2 iPSCs-CMs displayed a more immature phenotype with less organized myofibrils, enlarged sarcoplasmic reticulum cisternae and reduced number of caveolae. In a subsequent report by Binah’s lab [284], the group expanded the first study and investigated iPSC-CMs generated from CPVT1 and CPVT2 patients carrying the RyR2^R420Q^ and CASQ2^D307H^ mutations, respectively. The major findings were: (i) Ultrastructurally, CASQ2 and RyR2 mutated cardiomyocytes were less developed than control cardiomyocytes. (ii) While in control iPSC-CM isoproterenol caused positive inotropic and lusitropic effects, in the mutated cardiomyocytes isoproterenol was either ineffective, caused arrhythmias, or markedly increased diastolic [Ca^2+^]_i_. Importantly, positive inotropic and lusitropic effects were not induced in mutated cardiomyocytes. (iii) The effects of caffeine and ryanodine in mutated cardiomyocytes differed from control cardiomyocytes. These findings demonstrate that iPSC-CMs are useful for investigating the similarities/differences in the pathophysiological consequences of RyR2 versus CASQ2 mutations underlying CPVT1 and CPVT2 syndromes, respectively. Another study characterized the functional properties of iPSC-CMs originate from a CPVT1 patient carrying a novel RYR2 mutation [285]. The patient was presented with constant ventricular arrhythmic events under treatment with nadolol (a β-blocker) that were diminished during flecainide treatment. The generated iPSC-CMs expressed comparable quantities of excitation–contraction-coupling-related genes as the control cell line; however, Ca^2+^ handling properties were altered and abnormal in the CPVT1 iPSC-CMs. Consistent with the patient’s therapeutic in vivo response, treatment with nadolol during adrenergic stimulation achieved only slight rescue of the β-adrenergic-induced Ca^2+^ abnormalities. On the contrary, flecainide significantly enhanced Ca^2+^ handling properties including rectification of frequency and amplitude of Ca^2+^ waves and standardization of size, frequency and duration of Ca^2+^ sparks. Hence, patient-derived CPVT1 iPSC-CMs can reveal basic features of patient-specific drug responsiveness. In a related study, Pölönen et al. [286] investigated iPSC-CMs from skin biopsies of CPVT1 patients carrying exon 3 deletion (E3D) and L4115F mutation in *RYR2*. The main findings were: (i) APD_90_ of both E3D and L4115F CPVT1 iPSC-CMs was shorter than in control cells. E3D-CPVT iPSC-CMs had the shortest AP duration, lower AP amplitude, upstroke velocity and more depolarized diastolic potential than controls. (ii) CPVT1 iPSC-CMs exhibited increased incidence of DADs and EADs following epinephrine exposure, where E3D CPVT CMs had the most frequent DADs, EADs and tachyarrhythmia. (iii) Carvedilol abolished most of the arrhythmias in L4115F CPVT1 CMs. Finally, Itzhaki et al. [287] generated iPSC-CMs from a CPVT1 patient carrying a point mutation in the *RYR2* gene. Action potential measurements indicated no difference in AP properties between CPVT1 iPSC-CMs and control, except for arrhythmogenic tendency. Electrophysiological recordings indicated that DADs developed in 69% of the CPVT1 iPSC-CMs while only in 11% of control cells DADs were present. Induction of adrenergic stimulation using 5 µM forskolin or 1 µM isoproterenol significantly increased the frequency and amplitude of DADs in most of paced and non-paced CPVT1 iPSC-CMs, and led to development of triggered activity. In contrast, treatment with 10 µM flecainide significantly reduced or eliminated afterdepolarization events in all six tested CPVT1 iPSC-CMs. An inhibitor of the SR calcium ATPase pump which gradually depletes SR Ca^2+^ stores - thapsigargin, was used to examine the role of Ca^2+^ handling in the pathological generation of DADs. Application of thapsigargin onto forskolin-treated CPVT1 iPSC-CMs reduced afterdepolarization events post-pacing, implying the role of Ca^2+^ stores in DADs development. These results demonstrated the ability of CPVT1 iPSC-CMs to recapitulate the disease phenotype, the contribution of β-adrenergic signaling to increased propensity to arrhythmogenic events and the potential of using CPVT1 iPSC-CMs for specific drug-screening and evaluating suitable drugs.

#### 3.3.4. Metabolic Mutations

Metabolic cardiomyopathies include several inherited metabolic disorders which affect the heart and other organs, and occur as a consequence of disturbed energy production related to defects in glycogen, lipid and mucopolysaccharide metabolism. Systemic metabolic diseases, such as diabetes mellitus and alcoholism, may cause an acquired cardiomyopathy [288,289,290]. Metabolic cardiomyopathies, which include several inherited metabolic diseases in early childhood and affect the heart and other organs, occur because of disturbed energy production related to defects in glycogen, lipid and mucopolysaccharide metabolism. There is also a pathogenic pathway involved in cardiomyopathies associated with systemic metabolic diseases acquired during adulthood, such as diabetes mellitus and alcoholism [288,289,290]. Metabolic cardiomyopathies are also caused by mutations affecting metabolic enzymes such as PRKAG2 and LAMP2 [291,292]. At present, >100 different genes are known to be associated with non-ischemic cardiomyopathies or syndromes with cardiac involvement. It was found that the spectrum of affected genes and mutations partially overlaps between the different non-ischemic cardiomyopathies. In this section, we present two metabolic cardiomyopathies: Danon disease and familial Wolff–Parkinson–White (WPW).

##### Danon Disease

Danon disease is a rare X-linked dominant lysosomal disease, caused by fundamental deficiency of lysosome-associated membrane protein 2 (*LAMP2*) [293]. The *LAMP2* gene is located on the X chromosome and contains 9 exons, and different alternative splicing of the ninth exon generates three isoforms of the protein (LAMP-2A, -2B and -2C) [294]. Unfolded proteins recognized by the chaperone protein heat shock cognate 70 (Hsc70) and co-chaperons provide translocation of proteins signed for utilization directly into the lysosome via the receptor formed by LAMP-2A transmembrane protein [295]. Therefore, lack of LAMP2 leads to accumulation of autophagy material and often glycogen in cardiomyocytes. Major clinical features include skeletal and cardiac myopathy resulting in HCM, cardiac conduction abnormalities, mild intellectual difficulties, retinal disease and sudden cardiac death [291,296,297,298]. Early-onset fatal cardiomyopathy usually occurs in male patients, while female patients show a later onset and less severe clinical phenotype, which has been attributed to random inactivation of the *LAMP2* gene on the X chromosome [299]. Hashem et al. [300] generated iPSC-CMs from two patients with different *LAMP2* mutations and demonstrated impairment of autophagic flux, mitochondrial damage and increased apoptosis due to LAMP2 deficiency. In addition, these authors showed that the use of antioxidants may be beneficial to patients by neutralizing excessive free radicals from the oxidative stress. In another research by Ng et al. [301], Xi-chromosome reactivation of the silent LAMP2 allele, using a DNA methylation in iPSC-CMs from female Danon patients, improved its autophagy failure. Based on these and additional findings, the authors concluded that the iPSC-CM platform provides novel mechanistic and therapeutic insights into the contribution of random X chromosome inactivation to the disease phenotype in X-linked Danon disease. In a recent study, Yoshida et al. [302] generated mutant iPSC-CMs from clinically divergent monozygotic female twins with *LAMP2* mutations. This group found that compared with healthy control the mutant iPSC-CMs showed impairment in autophagosome maturation, in contrast to the previous study, but no difference in the apoptosis rate, and therefore concluded that apoptosis is less likely to be a key contributor to the pathologic mechanism of Danon disease. Recently, Chi et al. [303] generated iPSC-CMs from skin fibroblasts derived from *LAMP2* mutated patients and reported on a novel mechanism underlying Danon’s cardiomyopathy. The group identified the LAMP-2 isoform B (LAMP-2B) as required for autophagosome–lysosome fusion in human cardiomyocytes. Importantly, it was found that LAMP-2B functions independently of syntaxin 17 (STX17), a protein essential for autophagosome–lysosome fusion in non-CMs. Instead, LAMP-2B interacts with autophagy related 14 (ATG14) and vesicle-associated membrane protein 8 (VAMP8) through its C-terminal coiled coil domain (CCD) to promote autophagic fusion. Danon iPSC-CMs exhibit decreased colocalization between ATG14 and VAMP8, profound defects in autophagic fusion and mitochondrial and contractile abnormalities. These findings reveal a STX17-independent autophagic fusion mechanism in human cardiomyocytes, providing an explanation Danon’s cardiomyopathy and a potential therapeutic target.

##### Familial Wolff–Parkinson–White (WPW)

**Figure 6:**
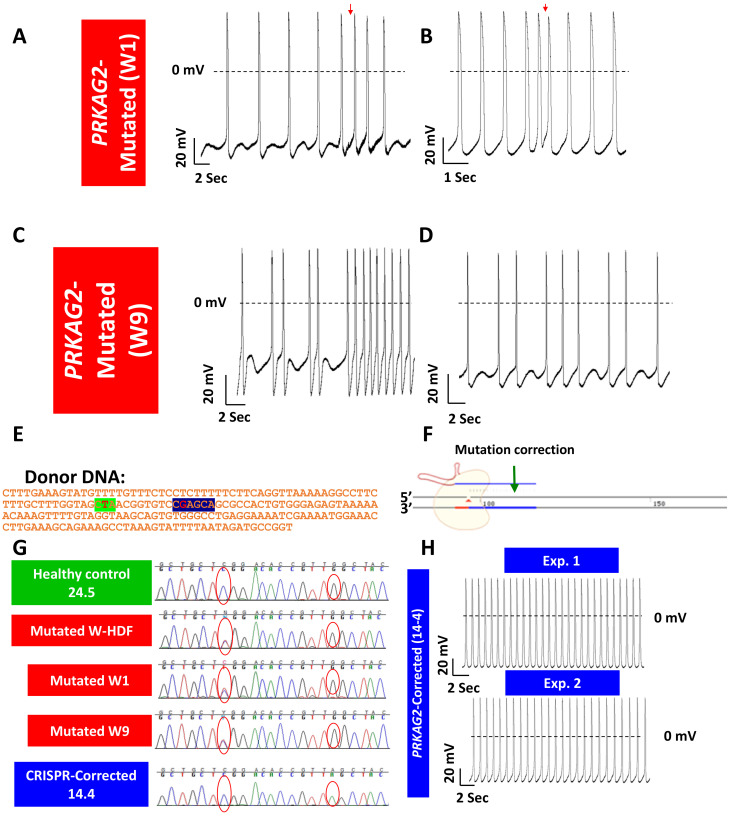
*PRKAG2*-mutated iPSC-CMs are arrhythmogenic: (**A**–**D**) Delayed afterdepolarizations (DADs) and triggered beats in the two mutated clones W1 (**A**,**B**) and W9 (**C**,**D**). Correcting the *PRKAG2* mutation using CRISPR technology abolished the arrhythmias in the patient’s iPSC-CMs: (**E**) ssODN was used as a donor DNA, in which PAM sequence (CCA) was replaced by a silent point mutation (CTA) (GCC/GCT = alanine). The two genetic modifications appear in red. (**F**) Representation of the designed strategy for point mutation correction using WT-Cas9 generated from Zhang Lab online tool (http://crispr.mit.edu). Green arrow represents mutation site in the chromosome, Cas9 interacting with gRNAs that are represented as blue-red bars (red part on the gRNA represents protospacer adjacent motif (PAM)). (**G**) Sanger sequencing analysis, in reverse mode, for the point mutation in the *PRKAG2* gene for the diseased clones (W-HDF, W1 and W9), WT healthy clone (24.5) and CRISPR-corrected clone containing silent substitution in the PAM site (14.4). Left and right red circles indicate point mutation and PAM sequence, respectively. (**H**) CRISPR-corrected iPSC-CMs display arrhythmia-free and normal firing pattern similar to healthy iPSC-CMs (two representative experiments) [90].

Familial WPW, characterized by pre-excitation, conduction abnormalities and hypertrophy, is caused by mutations in the *PRKAG2* gene encoding the γ-subunit of adenosine monophosphate kinase (AMPK) [304,305]. AMPK is one of the most critical metabolic regulators of carbohydrates and lipids in several tissues, including cardiac and skeletal muscles. AMPK plays a vital role in metabolism regulation [306], and increased AMP levels activate AMPK following a bioenergetics stress. Patients and animals with the *PRKAG2* gene mutation exhibit aberrant atrioventricular conduction associated with cardiac glycogen accumulation [90,307]. Thus, mutations in the *PRKAG2* gene in humans lead to HCM, autosomal dominant ventricular pre-excitation WPW, a progressive conduction system disease and vacuolar glycogen accumulation in cardiomyocytes [308]. Our group [90] recently investigated iPSC-CMs generated from a WPW patient carrying the R302Q mutation in the *PRKAG2* gene. We demonstrated that *PRKAG2*-mutated iPSC-CMs exhibit abnormal firing patterns, delayed afterdepolarizations, triggered arrhythmias and augmented beat rate variability. CRISPR correction eliminated the electrophysiological abnormalities, the augmented glycogen storage and cardiomyocyte hypertrophy (Figure 6). A similar study by Zhan et al. [309] reported that the *PRKAG2* mutation R302Q led to increased AMPK activity resulting in extensive glycogen deposition and cardiomyocytes hypertrophy. The researchers also reported that the adverse changes caused by the *PRKAG2*-R302Q mutation could be alleviated by small molecules inhibiting AMPK activity and rescued by CRISPR-Cas9 mediated genome correction.

### 3.4. Laminopathies

Laminopathies are a family of inherited diseases caused by mutations in the *LMNA* gene, which include, among others, Emery–Dreifuss muscular dystrophy type 2 and type 3, limb-girdle muscular dystrophy type 1B, dilated cardiomyopathy type 1A, Charcot–Marie–Tooth-disease type 2B1, familial partial lipodystrophy Dunnigan type and Hutchinson–Gilford progeria syndrome [310]. LMNA-related DCM is suggested to account for ~5–8% of familial DCM cases [192]. The disease is characterized by conduction system defects, arrhythmias, ventricular chamber dilation and impaired systolic function [311,312,313,314]. Although severe manifestations of the disease include life-threatening arrhythmias and end-stage heart failure [312,315], currently there is no definitive therapy targeting the specific cellular mechanisms underlying LMNA-related DCM. The *LMNA* gene encodes for the nuclear intermediate filament proteins A-type lamins; the two main products of this gene, lamin A and lamin C, result from alternative splicing and are expressed in most differentiated somatic cells [316,317]. Lamins are thought to be involved in a variety of cellular functions such as nuclear structural support, nucleo-cytoskeletal coupling, chromatin organization, gene expression and DNA repair [318,319,320]. In this regard, LMNA-mutant iPSC-CMs generated from patients may represent a useful tool for investigating mechanism-based treatments for the disease. Indeed, beneficial effects of such pharmacological modalities have been demonstrated in LMNA-mutant iPSC-CMs. In 2012, Siu et al. were the first to report on a iPSC-CMs model of LMNA-related DCM [321]. They generated iPSC-CMs from a patient with a R225X mutation in *LMNA* and from a patient with a frameshift mutation (contained a GCCA insertion at base 50) in *LMNA*. They showed that treatment with the mitogen-activated protein kinase kinase (MEK) 1/2 inhibitor, U0126, led to decreased percentage of cardiomyocytes with apoptosis mediated by electrical stimulation in LMNA^Frameshift/WT^ iPSC-CMs. In 2017, Lee et al. reported that application of PTC124, which promotes read through over premature stop codons, increased the levels of lamin A/C proteins and decreased the electrical stress-mediated nuclear blebbing and apoptotic population in LMNA^R225X/WT^ iPSC-CMs (with the UGA stop codon) [322]. In 2019, Lee et al. demonstrated that treatment of iPSC-CMs carrying a 348–349insG frameshift mutation in the LMNA gene (K117fs) with the Ca^2+^/calmodulin-dependent protein kinase II (CAMK2) inhibitor, KN93, led to reduced phosphorylated RYR2 (pRYR2) and phosphorylated CAMK2D (pCAMK2D) levels and to decreased percentage of cells with arrhythmic Ca^2+^ transients compared to treatment with vehicle or with the inactive analogue KN92 [323]. In addition, treatment with the platelet-derived growth factor receptor-β (PDGFRB) inhibitors, crenolanib and sunitinib, resulted in decreased percentage of cells with arrhythmic Ca^2+^ transients, decreased levels of pCAMK2D and pRYR2 and alterations in gene expression. Lastly, in 2019, Bertero et al. showed that application of the P/Q-type calcium channels inhibitors, ω-Conotoxin MVIIC and ω-Agatoxin TK, led to decreased field potential duration corrected for the beat period (FPDc) in LMNA^R225X/WT^ iPSC-CMs monolayers [324]. Additionally, treatment with the L-type calcium channel blocker, verapamil, resulted in decreased FPDc and spike amplitude in monolayers of LMNA^R225X/WT^ iPSC-CMs.

## 4. Summary

Drugs can cause cardiotoxicity through different cellular mechanisms including ion channel alterations, changes in intracellular Ca^2+^ handling and increased cardiomyocyte apoptosis. In turn, these changes cause increased risk of ventricular arrhythmias and depressed contractile function. The need to screen for ADRs, and specifically for cardiotoxicity, through the drug development process has been clear for years and different protocols have been developed to screen for such adverse reactions. The use of iPSC-CMs in the screening process has been recently implemented in the CiPA protocol in order to identify proarrhythmic drug effects with greater sensitivity and specificity. Cardiotoxicity, however, includes myopathic effects as well, which may have deleterious clinical consequences. Different iPSC-CM models have been developed for cardiotoxicity caused by anti-neoplastic drugs, potentially allowing identification of patients more susceptible to developing it. Similarly, various iPSC-CM models that have been described for specific acquired and inherited heart diseases. These may be able to help improve the detection of patients at risk for DIC and further studies are needed to test these models in the context of different drugs. The different iPSC-CM models for heart disease allow better understanding of specific disease mechanisms and may identify potential targets for drug intervention. Utilizing iPSC-CM disease models for drug development, using either known or newly developed drugs, opens up a new and exciting research field.

## Figures and Tables

**Table 1:** Drugs well-recognized to cause Torsades de Pointes. A listing of drugs and the strength of their association with Torsades de Pointes is shown at http://www.torsades.org.

Drug Group	Example
Antiarrhythmics	Amiodarone, Disopyramide, Dofetilide, Ibutilide, Procainamide, Quinidine, Sotalol
Antibiotics	Chloroquine, Clarithromycin, Erythromycin, Halofantrine, Pentamidine, Sparfloxacin, Antipsychotics, Chlorpromazine, Haloperidol, Mesoridazine, Pimozide, Thioridazine
Antinauseants/Antiemetic	Domperidone, Droperidol
Antineoplastic	Arsenic trioxide
Calcium channel blocker	Bepridil, Lidoflazine
Gastric promotility	Cisapride
Opiates	Methadone, Levomethadyl
Antihistamines	Terfenadine, Astemizole
